# Glutamate-Mediated Neural Alterations in Lead Exposure: Mechanisms, Pathways, and Phenotypes

**DOI:** 10.3390/toxics13070519

**Published:** 2025-06-21

**Authors:** Wagner A. Tamagno, Jennifer L. Freeman

**Affiliations:** School of Health Sciences, Purdue University, West Lafayette, IN 47907, USA; wtamagno@purdue.edu

**Keywords:** calcium signaling, EAAT, excitotoxicity, glutamate, neurotoxicity, NMDA, Pb, synaptic plasticity

## Abstract

Lead (Pb) is a pervasive neurotoxicant with well-documented detrimental effects on the central nervous system, particularly in vulnerable populations such as children. Despite historical recognition of its toxicity, Pb exposure remains a significant public health concern due to its environmental persistence, historical industrial use, and ongoing applications in modern technologies. This review focuses on the mechanisms by which Pb disrupts glutamatergic signaling, a critical pathway for learning, memory, and synaptic plasticity. Pb’s interference with glutamate receptors (ionotropic NMDA and AMPA, as well as metabotropic receptors), transporters (EAATs, VGLUTs, and SNATs), and metabolic pathways (glutamate–glutamine cycle, TCA cycle, and glutathione synthesis) are detailed. By mimicking divalent cations like Ca^2+^ and Zn^2+^, Pb^2+^ disrupts calcium homeostasis, exacerbates excitotoxicity, and induces oxidative stress, ultimately impairing neuronal communication and synaptic function. These molecular disruptions manifest cognitive deficits, behavioral abnormalities, and increased susceptibility to neurodevelopmental and neurodegenerative disorders. Understanding Pb’s impact on glutamatergic neurotransmission offers critical insights into its neurotoxic profile and highlights the importance of addressing its effects on neural function.

## 1. Introduction

Lead (Pb) is a pervasive and well-documented neurotoxicant whose toxicity has been extensively studied across a wide range of organisms, from cell culture [1,2] and *Caenorhabditis elegans* [3] to more complex biological models such as zebrafish [4,5], mice [6,7], and non-human primates [8] and humans [9,10]. Pb is also one of the oldest studied neurotoxicants in recorded history. The earliest documentation of its neurotoxic effects dates back to the first century, when the Greek physician Dioscorides noted exposure to Pb caused paralysis, delirium, gastrointestinal disturbances, and swelling, famously stating that “Pb makes the mind give way”.

However, if Pb toxicity has been recognized for so long and studied so extensively, the question remains as to why Pb exposure continues to pose a major global public health concern. The answer lies primarily in three key factors. First, Pb is a naturally occurring metal. Unlike synthetic chemicals that can degrade over time, metals cannot be created or destroyed by human activity; they are simply moved around the environment [11]. Humans extract Pb from natural deposits, incorporate it into manufactured products, and redistribute it, but they cannot alter the fundamental nature of the element itself [12]. However, its reactivity is highly dependent on its chemical form and speciation in biological media. Second, Pb has historically played a critical role in the advancement of human civilization. The so-called “Pb eras” of human history, spanning from the Bronze Age to Classical antiquity and into the modern era, reflect its widespread use in metallurgy, infrastructure, medicine, and even cosmetics [13]. During the Bronze Age, Pb was often alloyed with tin to create bronze, strengthening tools, weapons, and artifacts. In Roman times, Pb became essential for building aqueducts, plumbing systems, cookware, and cosmetics. Despite its known risks, Pb remained indispensable for centuries and continues to be used today, particularly in batteries, radiation shielding, and some types of ammunition [14]. Although its use in gasoline and paint has been phased out in many countries due to environmental and health concerns, Pb remains an important component of “green energy” initiatives, especially for energy storage technologies such as Pb-acid batteries [15]. Third, the indiscriminate and extensive use of Pb throughout history has left a lasting legacy of environmental contamination. Large amounts of Pb-containing waste persist in ecosystems worldwide, often improperly discarded. As a result, Pb continues to affect non-target organisms and re-enters the human food chain, with marginalized communities often disproportionately affected by environmental Pb pollution [16,17].

Beyond these three major reasons, several additional factors, such as ongoing industrial emissions, informal recycling practices, and legacy contamination, continue to contribute to global Pb exposure today [16,17]. This historical and environmental persistence raises another critical question on why Pb is particularly toxic to animals, especially humans. At a basic level, the answer appears straightforward: Pb is a non-essential metal with no known biological function. However, the biological story is more complex. Unlike essential metals like zinc (Zn), iron (Fe), and copper (Cu), which have evolved sophisticated systems for transport, storage, and elimination, Pb lacks dedicated physiological pathways [18]. Instead, Pb’s toxicity arises precisely because of its chemical properties: it mimics essential divalent cations like Ca^2+^ and Zn^2+^, allowing it to hijack their pathways and interfere with critical cellular processes [19,20]. Evolutionary pressures favored the safe handling of beneficial metals but offered no incentive to adapt to an inherently harmful element like Pb.

Inside the body, Pb acts as a “chemical saboteur,” disrupting biological systems by mimicking Ca^2+^ and Zn^2+^ [19]. It binds inappropriately to enzymes, receptors, and structural components, especially in tissues with high calcium dependence, such as the heart and brain. Historically, Pb poisoning has been clinically recognized through cardiopathies and neuropathies, the gold standards for diagnosing severe Pb toxicity [21]. Today, acute Pb poisoning is relatively rare. Modern exposure levels are generally much lower than the concentrations that historically triggered encephalopathy or cardiopathy [22]. Blood Pb levels (BLLs) above 70–100 μg/dL in children and 100–120 μg/dL in adults can cause severe neurological damage. At these levels, Pb crosses the blood–brain barrier, leading to encephalopathy. Cardiovascular risks are also heightened at relatively lower exposures. However, due to significant regulatory actions, such as those in the United States, high BLLs have decreased substantially [23]. For instance, according to the 2017–2018 National Health and Nutrition Examination Survey (NHANES), only 2.41% of Americans had BLLs at or below 3.16 μg/dL [24]. Furthermore, recognizing the particular vulnerability of children, the United States Centers for Disease Control and Prevention (US CDC) established a new Blood Pb Reference Value (BLRV) of 3.5 μg/dL. Alarmingly, approximately 2.5% of U.S. children aged 1–5 years still have BLLs at or above this threshold. Importantly, no level of Pb exposure is considered truly “safe,” especially for young children [25]. Children are particularly susceptible to Pb toxicity due to their immature physiology, greater hand-to-mouth behaviors, and higher relative intake of environmental contaminants (e.g., dust, soil, and paint chips). In addition, because their nervous systems are still developing, children are more vulnerable to disruptions in brain plasticity, which may lead to long-term cognitive, memory, and learning deficits. Some studies even suggest for every 10 μg/dL increase above the BLRV, there is an associated decline of approximately 2.6 IQ points [26].

In addition to the historical and industrial factors that have led to widespread Pb contamination, environmental injustice plays a significant role in the continued impact of Pb exposure today. Marginalized communities, often characterized by lower socioeconomic status and minority populations, are disproportionately affected by Pb contamination. These groups are more likely to live in areas with older housing; aging infrastructure; and proximity to industrial activities where Pb persists in the soil, water, and air. Limited access to resources, political representation, and healthcare further exacerbates the risks, leading to higher rates of Pb exposure and associated health effects in these populations [15,27]. This environmental injustice not only perpetuates health disparities but also highlights the urgent need for equitable policies and targeted remediation efforts to protect vulnerable communities from the long-term consequences of Pb exposure. In addition to the well-documented systemic and behavioral effects, recent advances highlighted Pb exposure can disrupt key molecular targets in the brain, particularly glutamatergic neurotransmission directly interacting with NMDA and AMPA ionotropic receptors. These mechanisms are explored in depth in the following sections.

Given Pb’s persistent environmental presence; its continuing usage in modern society; and its established role in cognitive decline, particularly among vulnerable populations, the pressing question becomes through what biological mechanisms does Pb induce neurobehavioral deficits at low exposure levels? Among the various pathways implicated, one that stands out is the glutamatergic system. Emerging evidence suggests Pb acts as a neurotoxicant by interfering with glutamatergic signaling, a key regulator of learning, memory, and synaptic plasticity [9,10,28,29]. Thus, this review focuses on how Pb impacts the glutamatergic system at different levels, from receptor function and transporter activity to astrocytic support mechanisms and the enzymatic cycling of glutamate within neurons. Understanding these mechanisms is crucial to unraveling how low-level Pb exposure triggers behavioral and cognitive impairments.

## 2. Overview of Glutamatergic Neurotransmission

Glutamate is the main excitatory neurotransmitter in the mammalian brain, playing a critical role not only in memory, learning, and cognition but also in a wide array of physiological functions that extend beyond classical neurotransmission. As a non-essential amino acid, glutamate can be synthesized endogenously, with its production dynamically regulated according to the organism’s metabolic demands [30].

During brain development, glutamate is essential for neuroplasticity, contributing to the brain’s ability to adapt structurally and functionally in response to environmental experiences [28]. Moreover, glutamate is involved in neuroendocrine secretion, mediating hormone release into the bloodstream [31]. Given its central role in numerous physiological processes, maintaining precise regulation of glutamate levels is critical. Both hyperactivity and hypoactivity of the glutamatergic system are implicated in various neuropathological conditions. Excessive glutamate can lead to excitotoxicity, overstimulation of neurons resulting in cellular damage, whereas deficits can impair essential synaptic functions. Alterations in glutamatergic neurotransmission are associated with several neuropsychiatric and neurodegenerative disorders, including schizophrenia [32], bipolar disorder [33], autism spectrum disorder [34,35]. Alzheimer’s disease [36], Parkinson’s disease [37], major depressive disorder [38,39], and amyotrophic lateral sclerosis (ALS) [38].

As with other neurotransmitters, glutamate synthesis occurs in the presynaptic neuron via multiple metabolic pathways. A primary route is the glutaminolysis pathway, where the enzyme phosphate-activated glutaminase (PAG) catalyzes the conversion of glutamine to glutamate and ammonia. Another important pathway involves glutamate dehydrogenase (GDH), which catalyzes the reversible oxidative deamination of glutamate to α-ketoglutarate and ammonia, thus linking glutamate metabolism with energy production. Additionally, the enzyme glutamine:2-oxoglutarate aminotransferase (GOGAT) facilitates the conversion of glutamine and 2-oxoglutarate into two molecules of glutamate, contributing to the neuronal glutamate pool [30].

Following synthesis, glutamate is packaged into synaptic vesicles by vesicular glutamate transporters (VGLUTs), specialized membrane proteins that exploit the proton gradient established by the vacuolar H+-ATPase (V-ATPase). The V-ATPase hydrolyzes ATP to pump protons into the vesicle lumen, generating a proton electrochemical gradient that drives glutamate accumulation within the vesicles, achieving high intra-vesicular concentrations [39]. Upon arrival of an action potential at the presynaptic terminal, voltage-gated calcium channels (VGCCs) open, allowing an influx of Ca^2+^ ions. This triggers vesicle fusion with the presynaptic membrane and the exocytotic release of glutamate into the synaptic cleft.

In the synaptic cleft, glutamate acts on two major classes of receptors: ionotropic and metabotropic. Among ionotropic receptors, the N-methyl-D-aspartate (NMDA), α-amino-3-hydroxy-5-methyl-4-isoxazolepropionic acid (AMPA), and kainate receptors are the most prominent. NMDA receptors are unique in their requirement for both glutamate binding and co-agonists (glycine or D-serine) for full activation, and they exhibit high calcium permeability. Their slower kinetics and voltage-dependent properties make them key mediators of synaptic plasticity, particularly in long-term potentiation (LTP) and long-term depression (LTD), processes fundamental to learning and memory. In contrast, AMPA receptors are responsible for mediating fast excitatory synaptic transmission. They predominantly generate rapid excitatory postsynaptic potentials (EPSPs) and are critical for the immediate excitatory drive at glutamatergic synapses. AMPA receptor trafficking and plasticity also underlie short-term and long-term modifications of synaptic strength [40].

In addition to ionotropic receptors, glutamate also acts through metabotropic glutamate receptors (mGluRs), which are G protein-coupled receptors (GPCRs) that modulate neuronal excitability and synaptic transmission through slower, longer-lasting signaling mechanisms. mGluRs are divided into three groups based on their sequence homology, signal transduction pathways, and pharmacological profiles: Group I (mGluR1 and mGluR5), Group II (mGluR2 and mGluR3), and Group III (mGluR4, mGluR6, mGluR7, and mGluR8). Group I mGluRs are primarily located postsynaptically and are typically coupled to Gq proteins, leading to activation of phospholipase C (PLC) and mobilization of intracellular calcium, which enhances excitatory neurotransmission and synaptic plasticity. In contrast, Group II and Group III mGluRs are generally found presynaptically and are coupled to Gi/o proteins, inhibiting adenylate cyclase activity and reducing cyclic AMP (cAMP) levels, which serves to inhibit neurotransmitter release and modulate synaptic strength. mGluRs play crucial roles in regulating synaptic plasticity, learning, memory, and neuroprotection, and their dysregulation has been implicated in a variety of neuropsychiatric and neurodegenerative disorders, such as anxiety, depression, schizophrenia, and Alzheimer’s disease [41,42,43,44].

Unlike acetylcholine, which undergoes enzymatic breakdown for signal termination, glutamate is primarily cleared from the synaptic cleft via reuptake mechanisms. This reuptake is crucial to prevent excitotoxicity and is mainly performed by astrocytes through a family of transporters known as excitatory amino acid transporters (EAATs). EAATs mediate the high-affinity, sodium-dependent uptake of glutamate from the extracellular space into glial cells and, to a lesser extent, into presynaptic neurons [45].

Once inside astrocytes, glutamate is rapidly converted into glutamine by glutamine synthetase (GS), an essential detoxification step given glutamate’s potential intracellular toxicity. Glutamine, a non-neuroactive amino acid, is then exported back into the extracellular space by sodium-coupled neutral amino acid transporters (SNATs). Astrocytic export predominantly involves SNAT3 and SNAT5 transporters, while neuronal reuptake is mediated by SNAT1 and SNAT2. Once reabsorbed by neurons, glutamine serves as a substrate for de novo glutamate synthesis via PAG or GOGAT activity, thus completing the glutamate–glutamine cycle critical for sustaining synaptic transmission [46].

Beyond its role in classical neurotransmission, glutamate also serves as a precursor for other critical molecules. In GABAergic neurons, glutamate is converted into gamma-aminobutyric acid (GABA) through the action of glutamate decarboxylase (GAD), thereby linking excitatory and inhibitory neurotransmission [47]. Moreover, glutamate contributes to cellular antioxidant defense by serving as a substrate for glutathione synthesis via the enzymes glutamate-cysteine ligase (GCL) and glutathione synthetase (GSS).

**Figure 1:**
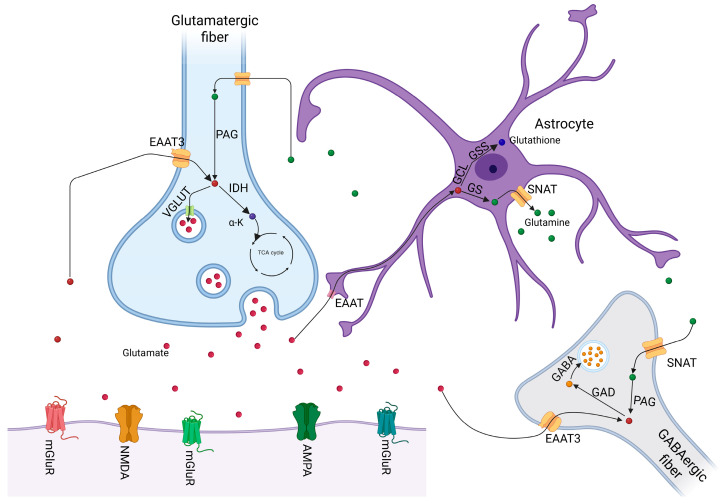
Glutamatergic signaling and main glutamate loops. Excitatory amino acid transporters (EAAT), phosphate activated glutaminase (PAG), isocitrate dehydrogenase (IDH), α-ketoglutarate (α-K), tricarboxylic cycle (TCA), vesicular glutamate transporters (VGLUT), glutamate decarboxylase (GAD), metabotropic glutamate receptors (mGluR), glutamine synthetase (GS), glutamate cysteine ligase (GCL), glutathione synthetase (GSS), sodium-coupled neutral amino acid transporters (SNAT). Created with BioRender.com.

An overview of the glutamatergic signaling pathways described above is depicted in Figure 1.

### Glutamate Dysregulation and Behavioral Impairments

As previously discussed, glutamatergic dysregulation is closely associated with impairments in cognition and learning, largely due to the critical role of glutamate in neuronal plasticity. Numerous studies have evaluated specific mechanisms by which glutamatergic dysfunction leads to measurable behavioral changes (Table 1). Behavior can be understood as a real-time manifestation of a functioning and dynamic central nervous system; however, it extends beyond neural activity alone. Behavioral outputs represent the integration of all physiological systems within the organism, processed and interpreted by the central nervous system. Moreover, behavioral manifestations are intrinsically dependent on environmental interactions and the individual’s interpretation of these external stimuli.

Considering the fundamental importance of glutamate in various brain functions and the inherent complexity of behavior, it becomes evident that alterations in glutamatergic signaling, depending on the exposure window, duration, and magnitude, can lead to distinct and detectable behavioral outcomes. For example, spatial memory impairments have been directly linked to the upregulation of hippocampal glutamate transporters in aged mice [29]. Similarly, in humans, reductions in the glutamate–glutamine cycling within the dorsal anterior cingulate cortex have been associated with impulsive decision making and gambling severity [48].

In addition, numerous dementia-related disorders, such as Alzheimer’s disease, have been closely connected to dysregulated glutamate levels, further emphasizing the neurotransmitter’s central role in neurodegeneration [49,50]. Neurodevelopmental disorders involving social interaction deficits, such as autism spectrum disorders, have also been linked to glutamatergic alterations [51,52]. In zebrafish models, disruptions in brain glutamate levels have been correlated with social impairments and increased anxiety-like behaviors [53,54].

Furthermore, dysregulation of glutamatergic signaling, particularly within the prefrontal cortex, has been consistently associated with the manifestation of schizophrenia-related symptoms and cognitive dysfunctions [32,55]. Supporting this, a pilot study in humans demonstrated a direct association between glutamate–GABA cycling disruptions and the severity of schizophrenia symptoms [56].

Taken together, these findings highlight that disruptions in the glutamatergic system consistently manifest as behavioral alterations across a wide spectrum of cognitive, emotional, and social domains. It is important to emphasize that the specific pathological outcomes depend not only on the brain region and functions affected but also vary based on species-specific neurobiology, environmental factors, and the developmental timing of the insult. Understanding these nuances is critical for elucidating the role of glutamate in health and disease.

## 3. Pb as a Neurotoxicant Targeting Glutamatergic Signaling

Integrating the concepts discussed thus far, it becomes evident Pb neurotoxicity frequently targets glutamate-related pathways, resulting in behavioral manifestations that are classically associated with Pb exposure, such as cognitive impairments and memory deficits (Table 1). Pb not only affects glutamate directly or its closely associated pathways, such as the glutamate–glutamine cycle and the glutamate–GABA cycle, but may also interfere with alternative regulatory pathways that indirectly influence glutamatergic homeostasis. These disruptions ultimately lead to glutamate dysregulation, impairing synaptic function and neuronal communication.

In this section, we will focus individually on distinct pathways and molecular components affected by Pb exposure that are either directly related to glutamatergic signaling or demonstrate a functional relationship with glutamate dysregulation. Whenever possible, we will aim to integrate each specific molecular or cellular dysregulation with a corresponding classical behavioral manifestation, establishing a direct link between central nervous system alterations in glutamatergic signaling and the resulting behavioral repertoire. This approach will allow for a comprehensive understanding of how Pb-induced glutamate dysfunction translates into observable neurobehavioral impairments.

It is also an important consideration to be stated in interpreting the outcomes of Pb toxicity studies, particularly in cell-based systems, is the speciation and solubility of Pb compounds. Pb salts such as Pb acetate can form insoluble complexes in standard culture media containing phosphate or sulfate ions, significantly affecting the bioavailable fraction of Pb^2+^. This has critical implications for dose–response interpretations and reproducibility [57,58].

### 3.1. Calcium Dysregulation and Glutamate Excitotoxicity Induced by Pb

A classical mechanism of Pb^2+^ toxicity is mimicking Ca^2+^, by which Pb^2+^ can aberrantly enter neurons through VGCCs [59] or bind to calcium-sensing and calcium-dependent proteins [60,60]. Due to its similar ionic radius and charge, Pb^2+^ can compete with or replace Ca^2+^ at critical regulatory sites [20], disrupting intracellular calcium homeostasis. Once internalized, Pb^2+^ leads to an abnormal increase in intracellular calcium levels, either by promoting excessive calcium influx [61] or by impairing calcium buffering and sequestration systems, such as the endoplasmic reticulum and mitochondria [62,63].

This dysregulation of intracellular Ca^2+^ signaling critically affects glutamatergic neurotransmission. Proper glutamate release from presynaptic terminals is tightly dependent on finely tuned calcium transients; however, Pb^2+^ exposure alters this process, leading to asynchronous, excessive, or insufficient glutamate release into the synaptic cleft [64]. Moreover, Pb^2+^ can sensitize or directly modulate glutamate receptors, particularly NMDA receptors, further disturbing calcium influx into the postsynaptic neuron [65].

The combined effects of increased presynaptic glutamate release and postsynaptic receptor hyperactivation promote a pathological process known as glutamate excitotoxicity. In excitotoxicity, the excessive stimulation of glutamate receptors, especially NMDA and AMPA receptors, lead to a sustained and uncontrolled calcium influx into the postsynaptic neuron. Elevated intracellular Ca^2+^ levels trigger downstream neurotoxic cascades, including activation of proteases (e.g., calpains), generation of reactive oxygen species (ROS), mitochondrial dysfunction, and initiation of apoptotic pathways [66].

Pb^2+^-induced calcium dysregulation thus not only compromises synaptic transmission and plasticity but also sets the stage for long-term neurodegenerative processes through excitotoxic injury. The persistent excitotoxic state can lead to synaptic loss, dendritic spine retraction, and neuronal death, which are key neuropathological substrates underlying cognitive and behavioral impairments observed after Pb^2+^ exposure [67,68,69].

Finally, Pb^2+^ trigger the inhibition of voltage-sensitive calcium channels (VSCCs) by blocking the L-type and other VSCCs, reducing Ca^2+^ influx necessary for neurotransmitter release (e.g., glutamate) and synaptic plasticity [70,71]. This inhibition disrupts presynaptic Ca^2+^ dynamics, impairing activity-dependent processes like LTP [68]. Internal high doses of Pb^2+^ displaces Ca^2+^ in calcium-binding proteins (CaBPs), for example, calmodulin and synaptotagmin with higher affinity than Ca^2+^, altering their function [19,20,72]. Pb^2+^-bound calmodulin fails to activate Ca^2+^/calmodulin-dependent kinases (CaMKII/IV), critical for memory consolidation [19].

### 3.2. Receptor-Mediated Glutamatergic Neurotoxicity by Pb

#### 3.2.1. Ionotropic Glutamate Receptors (NMDA and AMPA)

Pb^2+^ may be considered a non-competitive antagonist of N-methyl-D-aspartate receptors (NMDARs), which are critical for glutamatergic neurotransmission and essential processes such as synaptic plasticity, learning, memory, and LTP [73,74]. By blocking calcium influx through NMDARs, Pb^2+^ disrupts downstream signaling cascades, including the MAPK pathway, calcium/calmodulin-dependent protein kinase II (CaMKII), and CREB phosphorylation [64,75,76]. These disruptions impair the induction and maintenance of LTP and contribute to cognitive dysfunction. Pb^2+^ particularly affects GluN2A-containing NMDARs, binding to their Zn^2+^ regulatory sites with high affinity and suppressing their function in a voltage-independent manner [77,78,79]. Electrophysiological studies have shown that Pb^2+^ preferentially inhibits GluN2A subunits, leading to altered NR1 splice variant expression and reduced GluN2A levels in the hippocampus [68,77,80,81]. Additionally, Pb^2+^ exposure downregulates synaptic proteins and interferes with brain-derived neurotrophic factor (BDNF) signaling, further impairing synaptogenesis and neuronal survival [42,82]. Developmental Pb^2+^ exposure has also been linked to increased apoptosis, particularly during critical periods of brain formation, contributing to long-term neurodevelopmental abnormalities in both mammalian and zebrafish models [75].

Apparently less affected by Pb^2+^, AMPA receptors may be indirectly affected by it specially because of their interference with Zn^2+^. AMPA receptors are a subtype of ionotropic glutamate receptors, which are critical mediators of fast excitatory synaptic transmission in the central nervous system. Upon glutamate binding, AMPARs facilitate rapid sodium influx, resulting in depolarization of the postsynaptic membrane and initiation of EPSPs, which can ultimately trigger action potentials [83]. These receptors are primarily responsible for the initial phase of glutamate-induced synaptic activation, preceding the slower, voltage-dependent activation of NMDA receptors. Zn^2+^, co-released with glutamate during synaptic activity, exerts a modulatory effect on AMPARs in a concentration-dependent manner. At lower concentrations, Zn^2+^ enhances AMPAR function, potentiating synaptic responses, while at higher concentrations (typically >100 μM), it inhibits receptor activity, potentially acting as a negative feedback regulator [84,85]. This dual role suggests that Zn^2+^ serves as an important endogenous neuromodulator of glutamatergic signaling. Moreover, the regulation of Zn^2+^ homeostasis by metallothionein-3 (MT-3) may influence synaptic efficacy and neuronal excitability by modulating AMPAR and kainate receptor activity. These dynamic interactions position AMPARs at the core of synaptic integration and plasticity, with their function tightly regulated by both neurotransmitter binding and metal ion modulation.

#### 3.2.2. Metabotropic Glutamate Receptors (mGluRs)

mGluRs, particularly mGluR1 and mGluR5, are G-protein-coupled receptors that modulate excitatory neurotransmission, synaptic plasticity, and intracellular signaling cascades through activation of phospholipase C (PLC) and the resulting production of inositol trisphosphate (IP_3_) and diacylglycerol (DAG), ultimately leading to calcium release from intracellular stores and activation of protein kinase C (PKC). These receptors are activated by glutamate binding at the extracellular domain and are also sensitive to extracellular calcium, which can act as an allosteric modulator [86]. Emerging evidence suggests that mGluRs, especially mGluR5, are sensitive to developmental Pb²⁺ exposure. Pb has been shown to decrease mGluR5 mRNA and protein expression in a dose-dependent manner, impairing LTD and attenuating mechanisms of synaptic plasticity critical for learning and memory [87]. Pb^2+^ replace Ca^2+^ in PKC activation and calmodulin (CaM) interactions, thereby disrupting downstream signaling cascades essential for mGluR function [19,88]. Although mGluR1 and mGluR5 appear susceptible to Pb^2+^-induced modulation, some isoforms, such as mGluR3 and mGluR7, seem less affected, suggesting subtype-specific vulnerability [87]. Collectively, these findings highlight mGluRs as critical targets in Pb^2+^-induced neurotoxicity, particularly through disrupted calcium-dependent signaling and receptor phosphorylation dynamics.

Disruptions in glutamatergic signaling at the level of NMDA, AMPA, and mGluRs have profound implications for synaptic plasticity, learning, and behavior. Pb^2+^ exposure impairs NMDA receptor function by interfering with its redox and voltage-dependent regulation, leading to reduced calcium influx critical for LTP and memory formation [67]. Similarly, AMPA receptors, which mediate fast excitatory synaptic transmission, are differentially modulated by Zn^2+^ and Pb^2+^, with high concentrations causing receptor inhibition and altered postsynaptic excitability. At the metabotropic level, Pb^2+^-induced downregulation of mGluR5 expression and interference with mGluR1/Ca^2+^-dependent signaling cascades further compromise synaptic plasticity mechanisms such as LTD and intracellular calcium signaling. These molecular and functional alterations converge to impair key neural processes involved in cognition, learning, and emotional regulation. Behaviorally, such disruptions manifest as anxiety-like phenotypes, reduced locomotion, and impaired decision making in animal models, including zebrafish, which mirror aspects of human neurobehavioral deficits following developmental Pb exposure. This integrated evidence underscores the central role of glutamatergic dysfunction in mediating Pb^2+^-induced neurobehavioral toxicity [50,54,75,89,90,91].

### 3.3. Transporter-Mediated Disruption of Glutamate Homeostasis

#### 3.3.1. Excitatory Amino Acid Transporters (EAATs)

Pb can disrupt glutamate homeostasis through the modulation of EAATs, particularly EAAT3 and EAAT1. EAAT3 and EAAT1, primarily expressed in neurons, play a crucial role in the reuptake of extracellular glutamate, thereby preventing excitotoxic accumulation in the synaptic cleft [45]. Evidence from rodent studies indicates that Pb exposure increases EAAT3 protein expression, potentially as a compensatory response to elevated extracellular glutamate levels [92]. However, this upregulation may not be sufficient to counteract the excessive glutamate release induced by Pb-related oxidative stress and neuroinflammatory signaling. Moreover, Pb exposure has been associated with increased production of quinolinic acid (QA), a neurotoxic metabolite of the kynurenine pathway and a known NMDA receptor agonist [93]. QA can inhibit astrocytic glutamate uptake; stimulate neuronal glutamate release; and is itself transported into neurons via EAAT3, exacerbating excitotoxicity. The convergence of Pb-induced QA elevation, astrocytic dysfunction, and EAAT3-mediated glutamate accumulation creates a vicious cycle of NMDA receptor overactivation, oxidative damage, and neuronal loss. Thus, Pb disrupts glutamate homeostasis not only by altering transporter expression but also by facilitating the buildup of neurotoxic metabolites that act through glutamatergic pathways.

In addition to redox-sensitive modifications, increasing evidence suggests EAAT dysfunction in the context of Pb exposure may involve complex interactions between oxidative stress, calcium signaling, and astrocyte–neuron communication. Pb has long been recognized to interfere with calcium homeostasis by mimicking or displacing Ca^2+^ at various binding sites, including those involved in synaptic activity and intracellular signaling cascades. These disruptions may influence glutamate transporter expression and function indirectly, as calcium-dependent pathways, such as those mediated by protein PKC, calcium/calmodulin-dependent protein CaMKII, and calcineurin, play critical roles in regulating EAAT trafficking and activity [77,94]. Furthermore, Pb-induced oxidative stress may result in the direct modification of redox-sensitive cysteine residues on EAAT1 and EAAT2, impairing their conformational integrity and reducing transport efficiency, even in the absence of transcriptional changes [95].

Differential regulation of EAAT1 and EAAT2 under Pb exposure has been documented in several experimental models, suggesting transporter-specific vulnerabilities. While EAAT1 (GLAST) may exhibit some compensatory upregulation in response to increased extracellular glutamate or gliotransmitter feedback, EAAT2 (GLT-1) appears particularly susceptible to downregulation. This is consistent with findings that GLT-1 expression is tightly regulated by neuron-derived factors such as brain-derived neurotrophic factor (BDNF) and transforming growth factor-alpha (TGF-α), both of which may be disrupted by Pb-induced neurotoxicity [82,96]. These growth factors exert transcriptional control over GLT-1 through intracellular signaling cascades involving PI3K/Akt and NFκB pathways, both of which are known to be perturbed by Pb. Accordingly, Pb exposure during critical periods of brain development could compromise the trophic support necessary for the proper expression of GLT-1.

Moreover, Pb has been shown to impair mitochondrial function and reduce ATP availability, which could indirectly suppress EAAT activity by compromising the Na^+^ gradient required for glutamate uptake [97]. As EAATs are Na^+^-dependent co-transporters, any disturbance in ion homeostasis or energy metabolism is likely to exacerbate extracellular glutamate accumulation and excitotoxicity. In this context, the observed downregulation of EAAT2 may not only reflect transcriptional suppression but also post-translational mechanisms related to energy deficits and transporter mislocalization.

Another layer of complexity arises from the impact of Pb on neuron–glia interactions. Structural alterations in astrocytes, synaptic terminals, and dendritic spines have been reported following developmental Pb exposure, along with reduced synaptic density and impaired neurotrophic signaling [91,98]. These changes may diminish the reciprocal signaling required for maintaining astrocytic glutamate clearance capacity. Furthermore, early-life Pb exposure has been associated with epigenetic modifications, including promoter methylation and histone acetylation changes in genes encoding glutamate transporters, potentially leading to long-term transcriptional repression [8,89,94].

Finally, the inflammatory response triggered by Pb exposure may further compromise EAAT function. Pb has been shown to activate astrocytes and microglia, leading to the release of pro-inflammatory cytokines such as interleukin-1β (IL-1β) and tumor necrosis factor-alpha (TNF-α) [99]. These cytokines are known modulators of EAAT expression and may downregulate glutamate uptake capacity through both transcriptional and post-translational mechanisms.

Taken together, the available evidence indicates that Pb-induced EAAT dysfunction is likely to be multifactorial, involving oxidative stress, impaired calcium and energy homeostasis, altered neurotrophic support, disrupted neuron–glia communication, and inflammation. These converging mechanisms may underlie the persistent excitotoxic environment and contribute to the long-term neurodevelopmental deficits observed following early-life Pb exposure.

#### 3.3.2. Sodium-Coupled Neutral Amino Acid Transporters (SNATs)

While the role of EAATs in Pb-induced neurotoxicity has been relatively well explored, much less is known about the potential effects of Pb on SNATs, particularly members of the SLC38 family such as SNAT1 (SLC38A1) and SNAT2 (SLC38A2). These transporters play critical roles in glutamate–glutamine cycling by mediating the uptake of glutamine into neurons, thereby replenishing the glutamate pool necessary for synaptic transmission [100]. Although no direct studies to date have assessed the impact of Pb on SNAT expression or function, it is plausible that Pb may interfere with these transporters via similar mechanisms observed for EAATs, such as oxidative stress, altered calcium signaling, and disrupted astrocyte–neuron communication. SNAT1 and SNAT2 are known to be sensitive to intracellular signaling pathways, including those involving mTOR, MAPK, and PKC, many of which are perturbed by Pb exposure [101].

Disruption of SNAT-mediated glutamine transport could have downstream effects on neurotransmitter homeostasis, particularly under conditions of elevated extracellular glutamate where compensatory glutamine uptake becomes essential. Moreover, SNAT function depends on transmembrane Na⁺ and H⁺ gradients, both of which may be destabilized by Pb-induced mitochondrial dysfunction and ionic imbalance. Given the importance of glutamine uptake for synaptic plasticity, memory consolidation, and neuroprotection, further investigation into the potential vulnerability of SNATs to Pb toxicity is warranted. Future studies examining SNAT expression patterns, transport activity, and regulatory signaling under Pb exposure could provide valuable insights into additional mechanisms contributing to excitotoxicity and cognitive impairment.

### 3.4. Glutamate Release Machinery Disruption by Pb

#### 3.4.1. Vesicular Glutamate Transporters (VGLUTs)

The presynaptic release of glutamate, a critical step in excitatory neurotransmission, relies heavily on the proper function and expression of vesicular glutamate transporters (VGLUTs), which are responsible for packaging cytoplasmic glutamate into synaptic vesicles. While several studies have revealed the vulnerability of monoaminergic vesicular transporters to Pb exposure [28], VGLUTs appear to exhibit a more complex and nuanced response [102].

Immunohistochemical analyses have shown that, in contrast to the reduced expression of vesicular monoamine transporter 2 (VMAT2), synaptophysin, and serotonin markers in Pb-exposed brains, VGLUT1 levels often remain unaffected in specific brain regions such as the lateral superior olive (LSO) and the brainstem [28]. However, this preservation of VGLUT expression does not necessarily indicate preserved glutamatergic function. Other studies have reported that Pb exposure during synaptogenesis impairs vesicular release dynamics and presynaptic plasticity, as evidenced by decreased expression of key proteins like synapsin (Syn) and synaptobrevin (Syb), which interact with VGLUT-containing vesicles to facilitate docking and fusion [103].

Notably, Pb exposure has been associated with a significant reduction in the number of VGLUT1-positive synaptic elements that co-express Syn, suggesting that although transporter expression per se may remain stable, the synaptic machinery required for effective glutamate release is functionally compromised [102]. These alterations stem from Pb-induced disruptions in NMDA receptor-dependent BDNF signaling or oxidative stress pathways, which are known to influence presynaptic protein expression and synaptic integrity. Together, these findings highlight a potential dissociation between vesicular transporter presence and functional neurotransmitter release under Pb neurotoxicity, underscoring the need for further studies that investigate not only VGLUT expression but also its functional integration within the presynaptic release apparatus.

#### 3.4.2. Synaptic Vesicle Docking and Exocytosis Machinery

Beyond SNARE proteins, the efficient docking and exocytosis of synaptic vesicles involve a highly orchestrated network of presynaptic proteins, including Munc13, Munc18, complexin, and synaptotagmin, many of which are tightly regulated by calcium dynamics [104]. Pb exposure during neurodevelopment appears to disrupt this exocytic machinery at multiple levels. Reductions in synaptotagmin, a calcium sensor critical for synchronous vesicle release, have been implicated in the slowed kinetics of neurotransmitter exocytosis in Pb-exposed neurons [90,105].

Furthermore, Pb-induced dysfunction of synapsin and synaptophysin not only suggests compromised vesicle mobilization from reserve pools but also points to impaired docking at the active zone [103,106]. Imaging studies demonstrate that while the number of presynaptic contact sites may remain unaffected or even increase following Pb exposure (e.g., increased bassoon immunoreactivity), the quality and functionality of these sites are diminished, as evidenced by decreased vesicular protein co-localization and a decline in fast-releasing vesicle populations [102]. These alterations reflect a decoupling between structural synaptogenesis and functional maturation of the presynaptic terminal. Notably, the interaction of Pb with calcium-binding proteins and its interference with intracellular calcium buffering systems may further exacerbate these impairments, given that calcium is essential for vesicle docking, priming, and fusion. Altogether, Pb disrupts not only the presence of essential docking and fusion proteins but also the underlying calcium-dependent signaling that coordinates precise vesicle release, contributing to long-lasting deficits in excitatory neurotransmission.

#### 3.4.3. SNARE Complex

The SNARE (soluble NSF attachment protein receptor) complex plays a pivotal role in synaptic vesicle fusion with the presynaptic membrane, enabling neurotransmitter release into the synaptic cleft. This complex consists of vesicle-associated proteins such as synaptobrevin (VAMP), and target membrane proteins including syntaxin and SNAP-25 [107]. Emerging evidence indicates that developmental exposure to Pb disrupts the expression and function of key SNARE proteins, thereby impairing synaptic transmission [108]. For instance, Pb exposure has been associated with decreased levels of synaptobrevin, a critical vesicular SNARE required for fast vesicle fusion events [102]. This reduction likely hinders the proper assembly of the SNARE complex, compromising the speed and fidelity of glutamate release. While direct studies on Pb’s interference with the biochemical interactions within the SNARE complex remain limited, the observed decrease in vesicle-associated proteins such as synaptophysin and synapsin, both of which interact with SNARE elements, suggests a downstream effect on vesicle priming and fusion processes. Moreover, given that SNARE function is modulated by calcium signaling and that Pb can act as a calcium mimic, Pb^2+^ may competitively interfere with calcium-dependent vesicle fusion, further disrupting SNARE-mediated exocytosis. These mechanistic insights point to the SNARE complex as a vulnerable target of Pb neurotoxicity and warrant further research into how Pb affects its structural and regulatory components in glutamatergic neurons.

#### 3.4.4. Pb Disruption of Key Regulators of Vesicle Priming, Calcium Sensing, and Fusion

Efficient neurotransmitter release relies on a tightly regulated series of molecular events involving vesicle priming, calcium sensing, and membrane fusion, processes coordinated by proteins such as RIM, Synaptotagmin, SNAP-25, and Munc13. RIM (Rab3-interacting molecule) proteins play a central role in tethering primed vesicles at the active zone and anchoring VGCCs, thereby ensuring the spatial coupling between calcium influx and vesicle fusion. Synaptotagmin acts as the principal calcium sensor that triggers rapid neurotransmitter release upon calcium binding, while SNAP-25 forms part of the core SNARE complex, driving membrane fusion [104]. Developmental Pb exposure is known to disrupt this machinery at multiple levels. Pb’s interference with intracellular calcium dynamics, both by mimicking Ca^2+^ and altering calcium homeostasis, impairs the function of calcium-dependent proteins such as Synaptotagmin and RIM [19]. Munc13 functions as a vesicle priming factor, enabling the transition of synaptic vesicles into a release-ready state by facilitating SNARE complex assembly. Pb may hinder Munc13-mediated priming by disturbing upstream signaling cascades that regulate its activity (e.g., via DAG or PKC) [106], while reduced expression of SNAP-25 observed in some neurotoxicant models may compromise SNARE complex assembly and vesicle fusion [68]. Collectively, Pb-induced alterations to these proteins could result in fewer vesicles in a readily releasable state, reduced synchronization of release events, and overall decreased efficiency of excitatory synaptic transmission during critical neurodevelopmental windows.

### 3.5. Glutamate Metabolism and Cycling Disruption in Pb Exposure

#### 3.5.1. Tricarboxylic Acid (TCA) Cycle and Glutamate Biosynthesis

The equilibrium between glutamate and α-ketoglutarate is central to maintaining neurotransmitter glutamate levels and is tightly linked to mitochondrial energy metabolism through the tricarboxylic acid (TCA) cycle. Glutamate can be reversibly transaminated to α-ketoglutarate via glutamate dehydrogenase (GDH) or transaminases such as aspartate aminotransferase, allowing for both the clearance of excess synaptic glutamate and the replenishment of TCA cycle intermediates. Pb^2+^ exposure has been shown to interfere with this cycle at multiple levels. First, Pb^2+^ disrupts mitochondrial function, mainly due to Ca overload, including impairing key TCA cycle enzymes such as isocitrate dehydrogenase and α-ketoglutarate dehydrogenase, thereby reducing α-ketoglutarate availability for glutamate synthesis and energy production [109]. This mitochondrial dysfunction can also impair ATP-dependent processes critical for neurotransmitter cycling and synaptic maintenance. Furthermore, Pb²⁺ may directly inhibit GDH activity by altering its cofactor NAD^+^/NADH ratio when interacting with complexes II and III of the respiratory chain [63], leading to impaired glutamate catabolism. Such disruptions not only compromise glutamate clearance but also contribute to excitotoxicity due to glutamate accumulation. By targeting both glutamate metabolism and energy-generating pathways, Pb^2+^ creates a neurochemical imbalance that may underlie synaptic dysfunction and long-term neurodevelopmental deficits.

These Pb-induced impairments in mitochondrial metabolism suggest a critical disruption in the cross-talk between the TCA cycle and neurotransmitter homeostasis. Under normal physiological conditions, α-ketoglutarate serves as a key metabolic node linking energy production to glutamate synthesis. However, when Pb^2+^ compromises enzymes such as isocitrate dehydrogenase and α-ketoglutarate dehydrogenase, the pool of α-ketoglutarate is depleted, reducing substrate availability for glutamate production via transamination. Simultaneously, inhibition of GDH limits the reversible interconversion between glutamate and α-ketoglutarate, exacerbating the deficit. This breakdown in metabolic communication impairs the cell’s ability to maintain glutamate homeostasis, particularly during periods of high synaptic demand. The resulting imbalance affects both neurotransmitter cycling and the broader energy status of the neuron, reinforcing a vicious cycle of excitotoxicity and metabolic stress.

#### 3.5.2. Glutamate–Glutamine Shuttle

Pb exposure perturbs glutamatergic neurotransmission, partially through its effects on glutamate release and receptor dynamics within the glutamate–glutamine cycle. Pb consistently reduces stimulated glutamate release in several brain regions, including the hippocampus and cortex, likely through disrupted Ca^2+^-dependent mechanisms. This is paralleled by altered NMDA receptor function, where Pb acts as a noncompetitive antagonist, modifying subunit-specific receptor sensitivity and interfering with binding at sites typically regulated by zinc. Notably, developmental Pb exposure upregulates NMDA receptor subunits (e.g., GluN1/GluN2B), suggesting a compensatory response to reduced synaptic glutamate availability [102]. Pb also impairs astrocytic clearance of glutamate, a critical component of the glutamate–glutamine shuttle [110]. The resulting accumulation of extracellular glutamate may potentiate excitotoxicity, while astrocyte dysfunction compromises glutamine supply to neurons, ultimately disrupting the balance between excitatory and inhibitory signaling and contributing to cognitive and behavioral impairments.

#### 3.5.3. Glutamate–GABA Conversion

Pb neurotoxicity disrupts the glutamate–GABA cycle through multiple mechanisms affecting GABAergic transmission. Early studies revealed region-specific alterations in GABA receptor binding, with increased [^3^H]-GABA binding in the cerebellum but decreased binding in the striatum after chronic Pb exposure [64,111]. Pb impairs both GABA uptake and depolarization-evoked release in synaptosome preparations, likely through reduced expression of glutamate decarboxylase (GAD) and compensatory upregulation of GABA transporter 1 (GAT-1) [47,48,71]. Additionally, Pb induces spontaneous GABA release and alters GABA-B receptor properties, decreasing receptor affinity while modifying receptor density in a dose- and time-dependent manner [47]. Pb also inhibits VGCCs, diminishing tetrodotoxin-sensitive GABA release [70,112,113]. These cumulative effects suggest Pb impairs GABAergic homeostasis by interfering with GABA synthesis, transport, and receptor function, contributing to excitatory/inhibitory imbalance and neurobehavioral deficits.

#### 3.5.4. Glutamate–Glutathione Pathway and Oxidative Stress

Lastly, but critically, glutamate plays an essential role in the synthesis of glutathione (GSH), a tripeptide composed of glutamate, cysteine, and glycine. GSH is one of the most important non-enzymatic antioxidants in cells and is pivotal in maintaining redox homeostasis, detoxifying ROS, and protecting cellular macromolecules from oxidative damage. While the direct impacts of Pb on glutamatergic neurotransmission are well-documented, its indirect effects, particularly through oxidative stress and disruption of antioxidant systems, are equally significant but often underexplored.

Pb exposure has been widely associated with increased generation of ROS and reactive nitrogen species (RNS), mainly due to its interference with mitochondrial respiration and its ability to displace essential divalent cations (e.g., Ca^2+^ and Zn^2+^) [62,97]. This disruption impairs mitochondrial electron transport, leading to electron leakage and enhanced ROS production. Moreover, Pb inhibits antioxidant enzymes like superoxide dismutase (SOD), catalase (CAT), and glutathione peroxidase (GPx), exacerbating oxidative stress [63,114]. In response, cells increasingly rely on non-enzymatic antioxidants like GSH to neutralize ROS. However, sustained oxidative stress depletes intracellular GSH pools, and its resynthesis is highly dependent on glutamate availability. Glutamate uptake via EAATs is essential not only for neurotransmission termination but also for supplying substrates for GSH synthesis in astrocytes and neurons. Disruption of glutamate homeostasis by Pb can therefore impair GSH production, weakening the cellular defense system and amplifying oxidative damage. Studies have shown Pb exposure results in decreased GSH levels in neural tissue, concurrent with increased lipid peroxidation and protein carbonylation, indicating oxidative damage [115]. Furthermore, Pb can inhibit γ-glutamylcysteine synthetase, the rate-limiting enzyme in GSH synthesis, thereby compounding GSH depletion [116]. This creates a vicious cycle wherein Pb-induced glutamatergic disruption limits GSH synthesis, and reduced antioxidant capacity, in turn, intensifies Pb toxicity.

Taken together, the interplay between glutamatergic signaling and GSH synthesis under Pb exposure reveals a complex and bidirectional vulnerability, in which oxidative stress and excitotoxicity potentiate each other. Understanding this interaction is critical for elucidating the broader neurotoxic profile of Pb, especially during early developmental stages when the antioxidant system is not yet fully mature.

## 4. Conclusions

The evidence presented in this review underscores the profound neurotoxic impact of Pb on glutamatergic neurotransmission, highlighting its multifaceted disruption of synaptic function and plasticity. Pb interferes with glutamate homeostasis through several converging mechanisms, including antagonism of ionotropic receptors (notably NMDA receptors), dysregulation of key transporters (EAATs, VGLUTs), and disruption of calcium-dependent signaling pathways. These alterations result in excitotoxicity, oxidative stress, and impaired synaptic remodeling, all of which contribute to the cognitive and behavioral impairments frequently observed following Pb exposure.

**Figure 2:**
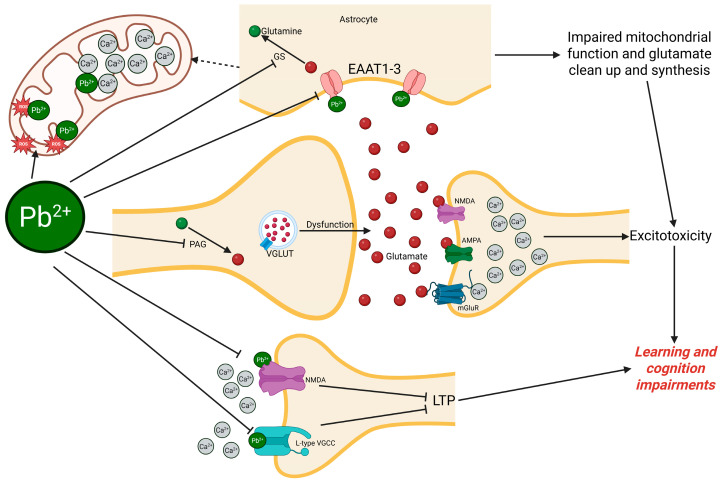
Mechanisms of Pb-induced glutamatergic impairment. Excitatory amino acid transporters (EAAT), glutamine synthetase (GS), phosphate-activated glutaminase (PAG), long-term potentiation (LTP), voltage-gated calcium channels (VGCC), N-methyl-D-aspartate transporters (NMDA), metabotropic glutamate receptors (mGluR), and vesicular glutamate transporters (VGLUT). Created with BioRender.com.

A central mechanism of Pb neurotoxicity is its calcium mimicry. Pb^2+^ competes with Ca^2+^ at both presynaptic and postsynaptic sites, disrupting glutamate release and receptor activation. This competition impairs LTP and other calcium-dependent processes critical for memory formation. Pb also induces receptor-specific dysfunction, including preferential inhibition of NMDA receptors, altered modulation of AMPA receptors by Zn²⁺, and downregulation of mGluRs, further compromising neural plasticity. A breakdown of the main glutamatergic target effects of Pb is summarized in Figure 2.

In addition to receptor-level effects, Pb exposure leads to significant transporter and metabolic dysfunction. Pb-induced oxidative stress and mitochondrial impairment reduce the efficacy of glutamate uptake through EAATs and hinder recycling via the glutamate–glutamine shuttle, thereby exacerbating extracellular glutamate accumulation and excitotoxicity. Notably, these disruptions are especially detrimental during neurodevelopment, when glutamatergic signaling is critical for neuronal maturation, synaptogenesis, and circuit formation. Early-life Pb exposure is thus strongly linked to long-term neurodevelopmental disorders and increased vulnerability to neurodegenerative diseases.

Importantly, this review highlights the bidirectional interplay between glutamatergic dysregulation and oxidative stress in Pb neurotoxicity. This interaction establishes a self-perpetuating cycle of neuronal damage that amplifies Pb’s toxic effects, particularly in immature or aging brains with compromised antioxidant defenses. Moving forward, research efforts should continue to investigate mechanisms of Pb neurotoxicity at doses relevant to historic and contemporary exposures to enhance confidence in mechanisms of glutamatergic dysregulation driving adverse neurological outcomes associated with Pb exposure. In addition, research efforts should prioritize therapeutic interventions aimed at restoring glutamate homeostasis, enhancing antioxidant capacity, and protecting calcium signaling pathways. Simultaneously, addressing environmental Pb contamination and enforcing equitable public health policies remain essential to prevent exposure, especially among vulnerable populations. By deepening our understanding of the molecular mechanisms by which Pb impairs glutamatergic signaling, we can better inform strategies for prevention, remediation, and neuroprotection, ultimately safeguarding brain health across the lifespan.

## Figures and Tables

**Table 1:** Glutamatergic targets affected by neurotoxicity, primary molecular effects, and associated behavioral phenotypes.

Glutamatergic Target	Primary Molecular Effect	Corresponding Behavioral Phenotype
Voltage-gated calcium channels (VGCCs)	Inhibition of calcium influx at presynaptic terminals, reducing vesicle fusion and glutamate release	Decreased synaptic transmission, impaired learning and memory
Vesicular glutamate transporters (VGLUTs)	Disruption of proton gradient and vesicular packaging, leading to reduced glutamate availability in vesicles	Impaired excitatory signaling, deficits in cognitive performance
NMDA receptors (ionotropic)	Inhibition of receptor function and altered subunit expression; dysregulated Ca^2+^ signaling and synaptic plasticity	Reduced long-term potentiation (LTP), memory impairments, increased anxiety-like behaviors
AMPA receptors (ionotropic)	Altered receptor trafficking and expression; imbalance in fast excitatory transmission	Impaired learning, increased impulsivity, reduced cognitive flexibility
Metabotropic glutamate receptors (mGluRs)	Dysregulation of Group I/II/III receptors; altered intracellular signaling cascades	Social deficits, affective disturbances, behavioral rigidity
Excitatory amino acid transporters (EAATs)	Impaired astrocytic reuptake of glutamate; extracellular glutamate accumulation and excitotoxicity	Neurodegeneration, hyperexcitability, anxiety-like and seizure-like behaviors
Glutamine synthetase (GS) in astrocytes	Reduced enzymatic activity, impairing glutamate detoxification and recycling	Long-term neurotransmitter imbalance, chronic excitotoxic effects, neurocognitive decline
Glutamate dehydrogenase (GDH) and GOGAT	Interference in metabolic conversion between glutamate and α-ketoglutarate	Energetic stress, altered synaptic efficacy, cognitive dysfunction
Glutamate–GABA conversion (via GAD)	Disrupted inhibitory–excitatory balance due to altered GABA synthesis from glutamate	Increased anxiety-like behavior, seizure susceptibility, altered stress reactivity

## Data Availability

No new data were created or analyzed in this study. Data sharing is not applicable to this article.

## Abbreviations

The following abbreviations are used in this manuscript:

AMPA	α-amino-3-hydroxy-5-methyl-4-isoxazolepropionic acid
BDNF	brain-derived neurotrophic factor
CaMKII	calcium/calmodulin-dependent protein kinase II
CREB	cAMP response element-binding protein
EAATs	excitatory amino acid transporter
GABA	gamma-aminobutyric acid
GPCRs	G-protein-coupled receptors
GSH	glutathione
LTD	long-term depression
LTP	long-term potentiation
mGluRs	metabotropic glutamate receptors
NMDA	N-methyl-D-aspartate
Pb	lead
ROS	reactive oxygen species
RNS	reactive nitrogen species
SNARE	soluble NSF attachment protein receptor
SNATs	sodium-coupled neutral amino acid transporters
TCA	tricarboxylic acid cycle
VGLUTs	vesicular glutamate transporters
VSCCs	voltage-sensitive calcium channels
